# Oncologists’ Reluctance to Use the Terms *Hope* and *Cure*: A Bibliometric Analysis of Articles From Two High-Impact Oncology Journals

**DOI:** 10.1093/jncics/pkaa065

**Published:** 2020-08-14

**Authors:** Benjamin W Corn, David Feldman, Lidia Schapira, David P Steensma, Charles L Loprinzi, Jiang Bian

**Affiliations:** p1 Shaare Zedek Medical Center, Jerusalem, Israel; p2 Santa Clara University, Santa Clara, CA, USA; p3 Stanford Cancer Institute, Palo Alto, CA, USA; p4 Dana Farber Cancer Institute, Boston, MA, USA; p5 Mayo Clinic, Rochester, MN, USA; p6 University of Florida, Gainesville, FL, USA

## Abstract

The words *cure* and *hope* are important terms in oncology, reflecting a balance of aspirations and realism for physicians and patients. Yet, some have suggested that oncologists are reluctant to use these terms. We tested this hypothesis by performing a bibliometric analysis of the frequency of use of these words in *JAMA Oncology (JAMA Oncol)* and the *Journal of Clinical Oncology* (*JCO*). The text of all articles in 3 categories—primary research, editorials, and narrative essays—appearing in *JCO* from 2000 to 2018 and in *JAMA Oncol* from 2015 to 2019 was analyzed. These analyses compared, across these categories, the proportion of articles containing the words *cure* and *hope*, as well as the proportion of total sentences containing these words. There were statistically significant differences in frequency of the use of the terms *cure* and *hope* as a function of the type of article published in the *JCO* and *JAMA Oncol* (2-sided *P* values ranging from .005 to <.001). Results were similar for both journals, with minor exceptions. Both *hope* and *cure* were used in a greater number of articles and sentences in the narrative and editorial categories than in primary research. Moreover, *hope* was used more often in narrative essays than in editorials. The relative reluctance to use these terms in more scientifically oriented original reports, despite concomitant improvements in oncologic outcomes, may reflect a bias worthy of future exploration.

During a 2018 keynote address (1) to the American Society of Clinical Oncology, Dr Norman Sharpless (director of the National Cancer Institute) observed, “An overarching worry of today’s oncologist has been the management of expectations. We don’t want to overpromise and give hope that is false … I think we have become scared to tell patients that we actually hope to cure them, and it may be time to examine how we communicate our efforts in this area.” We realize that it is rational—and even ethical—for physicians to avoid exaggerating possible benefits of treatment. However, it was sobering to hear Sharpless further suggest that oncologists are simply uncomfortable with words like *cure* and *hope* even though patients crave them.

If oncologists use these terms infrequently, such caution may not be entirely warranted. With regard to *cure*, data from the American Cancer Society indicate that survival has steadily improved for almost 30 years, for nearly all common malignancies (2). For instance, the overall annual cancer death rate dropped continuously from 1991 to 2017 by 29%. In the United States, approximately 2.5 million fewer people have died of cancer during the past 3 decades than would have died if cure rates had remained unchanged. But, have oncologists been willing to acknowledge this reality to their patients and among themselves?

*Hope*, for its part, is no longer an amorphous concept, having been rigorously investigated with validated tools during the same 3-decade period (3,4). Specifically, an operationalized model known as Hope Theory has emerged that conceptualizes hope as a goal-directed construct. Given that goals consist of anything that an individual desires to do, be, get, or experience, hope matters in virtually every context and stage of life. In its absence, patients are often beset by emotional distress and decreased ability to cope with physical symptoms, and healthcare providers may be at risk for burnout (5–7). Given Sharpless’ comments, however, it is important to note that hope need not be focused solely on goals related to cure or prolongation of life. In fact, it may be desirable to redirect patients diagnosed with malignant disease toward noncancer-related hopes that are important to them, even when cure is unlikely (8,9).

But, are oncologists indeed reluctant to use the terms *hope* and *cure*? To answer this question in a data-driven manner, we performed quantitative bibliometric analyses of trends in the published literatures of 2 high-impact oncology journals.

## Methods

The journals *JAMA Oncology* (*JAMA Oncol*) and *Journal of Clinical Oncology* (*JCO*) were selected for analysis not only because of their high impact but also because they both regularly publish articles in 3 categories: primary research (*Original Investigations* in *JAMA Oncol*; *Original Reports* in *JCO*), editorials (*Viewpoints* in *JAMA Oncol*; *Editorials* in *JCO*), and narrative essays (*Cancer Care Chronicles* in *JAMA Oncol*; *Art of Oncology* in *JCO*). In *JAMA Oncol*, narrative essays are defined (10) as “personal vignettes taken from wide ranging experiences in medicine,” and in *JCO*, such essays are intended to (11) “explore the experience of suffering from cancer or caring for people with cancer.” Editorials in *JAMA Oncol* are defined as “opinions that address any important topic in medicine, public health, research, discovery, prevention, ethics, health policy or health law.” A strict definition of editorials is not provided in the “Information for Authors” section of *JCO*. To avoid confusion, we will use the terms *primary research articles* (PR), *editorials* (ED), and *narrative essays* (NE) to refer to the above categories, regardless of journal.

We analyzed usage patterns of the words *hope* and *cure* in all articles appearing since the inception of these journals’ respective narrative sections. Accordingly, for *JCO*, we examined a 19-year period from 2000 to 2018 (12 604 articles) and, for *JAMA Oncol*, a 5-year period from 2015 through 2019 (759 articles). We obtained raw text of all articles in electronic format and performed a series of 2-proportion *z* tests using the StatsModels module (12) in Python. These analyses yielded comparisons across all 3 categories (PR, ED, NE) of the proportion of articles that mentioned *hope* or *cure* at least once in their text as well as the total number of sentences containing these words. All *P* values are 2-sided, with initial critical values for statistical significance set at .05. However, given that we performed multiple comparisons, Bonferroni correction was used, resulting in a corrected critical value for statistical significance of .008 for each set of analyses.

For reasons we discuss in detail below, we expected that a greater proportion of NE articles than PR or ED articles would mention the words *hope* and/or *cure*, and a greater proportion of ED than PR pieces would mention these target words. Because it is possible that articles in any of these categories could mention a target word but not use it frequently, we also analyzed the proportion of total sentences containing these words, expecting a parallel pattern.

## Results

Results were nearly identical for the 2 journals, with only minor exceptions. Table 1 portrays article-level statistics. As expected, in both journals, *hope* was mentioned at least once in the text of a greater proportion of NE than ED or PR articles and in a greater proportion of ED than PR articles. Also in both journals, *cure* was mentioned at least once in the text of a greater proportion of both NE and ED articles than PR articles (the NE-PR comparison was approaching statistical significance). Only in *JCO*, however, was *cure* used in a greater proportion of NE than ED articles. Findings were similar at the sentence level (Table 2).

**Table 1.** Article-level descriptive statistics and comparisons

Journal	Article category	No. of articles	No. (%) of articles mentioning *cure*	No. (%) of articles mentioning *hope*	Comparisons for each target word	*z* score	*P* ^a^
*Journal of Clinical Oncology*	NE	300	117 (39.0)	180 (60.0)	Cure: NE > ED	6.83	<.001
					Hope: NE > ED	16.35	<.001
	ED	1748	365 (20.9)	295 (16.9)	Cure: ED > PR	3.84	<.001
					Hope: ED > PR	12.34	<.001
	PR	10 556	1805 (17.1)	817 (7.7)	Cure: PR < NE	−9.80	<.001
					Hope: PR < NE	−30.91	<.001
*JAMA Oncology*	NE	43	10 (23.3)	18 (41.9)	Cure: NE = ED	0.07	.94
					Hope: NE > ED	3.26	<.001
	ED	164	39 (23.8)	30 (18.3)	Cure: ED > PR	3.00	.003
					Hope: ED > PR	2.82	.005
	PR	552	77 (14.0)	56 (10.1)	Cure: PR < NE	−1.66	.096
					Hope: PR < NE	−6.07	<.001

a Two-proportion *z* tests; 2-sided *P* values given. ED = editorial; NE = narrative essay; PR = primary research.

**Table 2.** Sentence-level descriptive statistics and comparisons^a^

Journal	Article category	No. of sentences	No. (%) of sentences with word *cure*	No. (%) of sentences with word *hope*	Comparisons for each target word	z score	*P* ^a^
*Journal of Clinical Oncology*	NE	31 444	298 (1.0)	719 (2.3)	*Cure*: NE > ED	13.22	<.001
					*Hope*: NE > ED	45.64	<.001
	ED	172 383	672 (0.4)	391 (0.2)	*Cure*: ED > PR	37.92	<.001
					*Hope*: ED > PR	39.65	<.001
	PR	4 335 756	3963 (0.09)	1406 (0.03)	*Cure*: PR < NE	−48.46	<.001
					*Hope*: PR < NE	−180.60	<.001
*JAMA Oncology*	NE	3466	25 (0.7)	66 (1.9)	*Cure*: NE = ED	0.91	.36
					*Hope*: NE > ED	8.82	<.001
	ED	9825	87 (0.9)	37 (0.4)	*Cure*: ED > PR	17.90	<.001
					*Hope*: ED > PR	8.80	<.001
	PR	119 456	133 (0.1)	96 (0.08)	*Cure*: PR < NE	-9.88	<.001
					*Hope*: PR < NE	-29.17	<.001

a Two-proportion *z* tests; 2-sided *P* values given. ED = editorial; NE = narrative essay; PR = primary research.

Of note, we also found evidence of diminishing use of the terms *hope* and *cure* over time (Tables 3 and 4) (2). Analyses evaluating this issue were only performed on articles from *JCO*, given the longer timespan of available data (2000-2018). Specifically, we divided the articles into 4 time periods and performed a series of Cochran-Armitage trend analyses to test for historical change. Although most of these analyses yielded non-statistically significant results , there were a number of exceptions to this rule. At the article level, the proportion of pieces containing the word *cure* statistically significantly decreased over time in both the NE (*P* = .004) and PR categories (*P* < .001). At the sentence level, the proportion of sentences containing the word *cure* statistically significantly decreased over time in both the ED (*P* < .001) and PR (*P* < .001) categories, as did the proportion of sentences containing the word *hope* in the PR category (*P* < .001). Thus, despite the improving trends in oncologic outcomes over time, professionals seem less likely to mention both *hope* and *cure* in published work.

**Table 3.** Article-level historical trends in the *Journal of Clinical Oncology*

Article category	Year	No. of articles	No. (%) of articles mentioning *cure*	No. (%) of articles mentioning *hope*
Narrative essay	2000-2004	66	35 (53.0)	35 (53.0)
	2005-2009	85	30 (35.3)	58 (68.2)
	2010-2014	87	31 (35.6)	49 (56.3)
	2015-2018	62	21 (33.9)	38 (61.3)
Editorial	2000-2004	280	74 (26.4)	42 (15.0)
	2005-2009	608	113 (18.6)	105 (17.3)
	2010-2014	541	121 (22.4)	104 (19.2)
	2015-2018	319	57 (17.9)	44 (13.8)
Primary research	2000-2004	2579	577 (22.4)	195 (7.6)
	2005-2009	3552	584 (16.4)	277 (7.8)
	2010-2014	3025	427 (14.1)	225 (7.4)
	2015-2018	1400	217 (15.5)	120 (8.6)

**Table 4.** Sentence-level historical trends in the *Journal of Clinical Oncology*

Article category	Year	No. of sentences	No. (%) of sentences with word *cure*	No. (%) of sentences with word *hope*
Narrative essay	2000-2004	8304	104 (1.3)	187 (2.3)
	2005-2009	9954	79 (0.8)	238 (2.4)
	2010-2014	7445	69 (0.9)	215 (2.9)
	2015-2018	5741	52 (0.9)	92 (1.6)
Editorial	2000-2004	27 988	158 (0.6)	70 (0.3)
	2005-2009	60 687	239 (0.4)	166 (0.3)
	2010-2014	49 882	230 (0.5)	153 (0.3)
	2015-2018	33 826	98/(0.3)	62 (0.2)
Primary research	2000-2004	966 541	1438 (0.2)	579 (0.06)
	2005-2009	1 489 180	1529 (0.1)	873 (0.06)
	2010-2014	1 246 093	1121 (0.09)	611 (0.05)
	2015-2018	633 942	506 (0.08)	275 (0.04)

## Discussion

Bibliometric analyses like those reported here are known and accepted methods for gauging trends in a field (13). Nonetheless, like any methodology, they are imperfect. For instance, it is difficult, if not impossible, to determine the exact meaning of every mention of *hope* and *cure* in the 13 363 articles represented in our analysis. For this, nearly prohibitively detailed qualitative analysis of every instance would be necessary. Moreover, alternative interpretations of our findings must be entertained. Does the relative absence of *hope* and *cure* from written communication indicate commensurate disinclination to speak these words with patients? Does the use of *hope* and *cure* differ between academic investigators—who author most articles—and practitioners who deliver care outside of academic settings? Do people have differing conceptions of the terms *hope* and *cure* (14)? As several authors have noted, despite attempts to define *cure* for patients with cancer, the term is used heterogeneously in the oncology literature (15,16). We see these as questions worthy of further investigation.

Placing our 3 article categories on a continuum of descending scientific content, authors appear less likely to use *cure* and *hope* in more empirically rigorous primary research articles than editorial or narrative pieces. These results may not seem surprising, although the implications are potentially far-reaching. Divergent explanations exist. First, *hope* and *cure* might not be taken seriously by oncologists and therefore not incorporated into primary research articles. Conversely, heightened respect could be attached to these terms, such that authors do not feel they are appropriate in manuscripts where primary research is reported. Or, it may simply be that *hope* and *cure* are often omitted from primary research articles because they are not designated as outcome measures in the investigations typically reported in these 2 journals. Another viewpoint is that the narrative essay—a genre that, by design, enables authors to be reflective—is likely to be enriched by concepts like *hope*, which people may consider meaningful but are reluctant to mention in more professional contexts, given the perception that they are overly abstract or sentimental.

Regardless, our findings are consistent with Dr Sharpless’ commentary about reluctance to use these terms despite improving survival rates for patients and the growing understanding of *hope* as a construct that can be rigorously measured and is not merely synonymous with the possibility of *cure* (2,3). In the future, we propose further exploration of this hypothesis in the literatures of related specialties such as geriatrics or intensive care and of other healthcare professions such as nursing or social work.

In the meantime, physicians caring for patients with cancer may wish to reacquaint themselves with the accomplishments of their colleagues (2) to appreciate the instances where cure is achievable while simultaneously broadening their definition of hope when cure is elusive. Perhaps the time has come to judiciously embrace the words *hope* and *cure*.

## Funding

This work was supported, in part, by the Pamm Gross Kahane Research Institute of Life’s Door.

## Notes

**Role of the funder:** The funder had no role in the writing of this commentary or the decision to submit it for publication.

**Conflicts of interest:** The authors have no conflicts of interest to disclose.
